# Editorial: Rising stars in food chemistry: insights on plant polyphenols and their polymers

**DOI:** 10.3389/fnut.2023.1225324

**Published:** 2023-06-13

**Authors:** Hongkai Zhu, Qunfeng Zhang, Youben Yu, Paul A. Kilmartin, Liang Zeng

**Affiliations:** ^1^Key Laboratory of Tea Biology and Resource Utilization, Tea Research Institute, China Academy of Agricultural Sciences, Hangzhou, China; ^2^Department of Tea Science, College of Horticulture, Northwest A&F University, Xianyang, China; ^3^School of Chemical Sciences, The University of Auckland, Auckland, New Zealand; ^4^Department of Tea Science, College of Food Science, Southwest University, Chongqing, China

**Keywords:** plant polyphenols, chemical property, biological activity, nature products, biosynthesis

Plant polyphenols enjoy an ever-increasing recognition because their regular consumption has been claimed to be beneficial for human health (1). Plant polyphenols are present and abundant in fruits, flowers, vegetables and derived foodstuffs and beverages, and are known for their strong antioxidant properties based on scavenging free radicals and reactive oxygen/nitrogen species (2). The antioxidant properties of plant polyphenols have often been highlighted as the fundamental chemical event that underlies their utility in reducing the risk of certain age-related degenerations and diseases, and they are associated with antibacterial, antitoxin, antiviral and antifungal properties (3). These active functions have created significant interest within industry, and plant polyphenols have been developed for a wide range of applications such as food supplements, packaging materials, and pharmaceutical and cosmetic additives (4). In addition, cultivation and processing can affect plant polyphenols composition, content and structure, even producing new polyphenol polymers. These changes are promising to form new bioactivities, functions and application areas.

Much recent research has been undertaken regarding the metabolism, chemical properties, functions, and application of plant polyphenols and their polymers. Researchers hope to delve into the effects of metabolic change and chemical properties, relevant to the bioactivity and functions of plant polyphenols and their polymers, considering the wide range of application possibilities in the food and pharmaceutical industries. This complements existing knowledge with new information on polyphenol chemistry, based not only on bioactivity and antioxidant mechanisms, but also on the relationship between structure and functions. This editorial aims to summarize the Research Topic studies involving how the structure of plant polyphenols affect bioactivity and function, and how to regulate the composition and content of polyphenols in foodstuff by cultivation and processing. It will broaden our knowledge of plant polyphenols and their polymers and prospects for health efficacy and industrial applications.

Ren et al. used *Eucommia ulmoides* leaves as a functional additive to develop a new sweet rice wine containing a variety of health efficacies. The new sweet rice wine presented a high level of activity in scavenging DPPH, superoxide anion, and hydroxyl radicals, compared to wines without added *E. ulmoides* leaves, as well as the inhibiting effects on α-amylase, α-glucosidase, and angiotensin-converting enzymes. These bioactivities indicated that the developed sweet rice wine may have potential antihypertensive, antihyperglycemic and antihyperlipidemic effects connected with the higher flavonoid contents.

Shan et al. investigated the influence of dynamic changes of non-volatile metabolites during the withering process of Longjing green tea on sensory quality. They found a significant and well-marked increase in catechin oxidative polymerization products during the withering process, including theasinensins and theaflavins. These polymers combined with malic acid, succinic acid, quinic acid, and coumaroylquinic produced large effects on the sensory quality of Longjing green tea. Further, Zhang et al. explored bitterness quantification and the sensory response mechanism of theasinensin A, which is a major group of catechin dimers mainly found in oolong tea and black tea. Theasinensin A was found to exhibit preference for affinity with the N-terminal of bitterness receptor protein TAS2R16, rather than with TAS2R13 and TAS2R14, and its dose-over-threshold value was significantly higher than caffeine in black tea infusion.

Pu et al. characterized the difference in flavonoid metabolites in chayote fruits during storage. It was confirmed that the exogenous application of phenylalanine during storage increased the total content of flavonoids and theaflavins by adjusting some flavonoid biosynthesis-related gene expression pathways. Luo et al. found that tea polyphenols with free amino acids, caffeine and soluble sugar presented regional variations in Matcha from different regions in China, as well as variation in volatile components. The differences among the tea samples from different regions is probably induced by the different soil conditions, as high-pH areas can limit the ability of tea plants to uptake nutrient elements, including nitrogen, phosphorus, potassium, zinc, copper etc. This results in a decline in biosynthesis in tea leaves and limits the production of tea polyphenols, amino acids, and other quality components (Li C.-L. et al.).

Catechins are the most important plant polyphenols and functional compounds in tea beverages (5). Li X.-X. et al. systematically and comprehensively reviewed the anticarcinogenic activities of tea catechins by analyzing the advanced epidemiological evidence and anticarcinogenic mechanism. Up to date, the explored anticarcinogenic mechanism of catechins include inhibiting the proliferation, growth and metastasis of cancer cells, scavenging free radicals, improving immunity, strengthening the effects of anticancer drugs, and regulating signaling pathways. These mechanisms of action could provide insights into the development of anticancer drugs and carriers based on catechins.

Together these seven reports cover interesting aspects relevant to plant polyphenols and their polymers in the context of biosynthesis, regional differences and processing effects, bioactivity, physicochemical properties and the application of polyphenols plants in food development. These works provide new insights into the application and development of plant polyphenols, bridging the gap between practical applications and theoretical research.

## Author contributions

HZ: conceptualization, writing—original draft, writing—review and editing, and supervision. QZ, YY, PK, and LZ: writing—review and editing and supervision. All authors contributed to the article and approved the submitted version.
